# Challenges and strategies for the internationalization of higher education in low-income East African countries

**DOI:** 10.1007/s10734-023-00994-1

**Published:** 2023-01-23

**Authors:** Mohammad Moshtari, Alireza Safarpour

**Affiliations:** 1 Faculty of Management and Business, Industrial Engineering and Management, Tampere University, Tampere, Finland; 2 Sharif Policy Research Institute, Tehran, Iran

**Keywords:** Internationalization of higher education, East Africa, Low-income countries, University, Sustainable Development Goals, COVID-19 pandemic

## Abstract

As it becomes more crucial to push the boundaries of science to develop new technologies and important global initiatives, internationalization can be instrumental in helping underdeveloped countries overcome challenges such as poverty, climate change, and educational inequalities. Higher education institutions have always faced challenges in the process of internationalization, which have occupied scholarly attention in recent decades, but little research has been conducted on the internationalization of higher education in less developed African countries. This qualitative study aims to shed light on the challenges of internationalization of higher education in low-income countries in East Africa. After reviewing the literature and interviewing academics, the obtained data were thematically analyzed. The results suggested 12 main challenges, which were classified into four major categories. The challenges include a lack of clear policies and guidelines; the inefficiency of the organizational structure of internationalization; financial, infrastructure, and equipment problems; weaknesses in scientific, skill, and language competences; cultural differences; non-reciprocal relationships; and a brain drain. Finally, strategies for responding to these challenges with regard to the internal and external environments of higher education institutions were proposed. Among the internal strategies of higher education institutions are the development of clear policies and visions, planning for the development of human resources, and sustainable budgeting for internationalization programs. External strategies emphasize the development of national policies and laws based on contextual and environmental conditions, as well as interaction and participation in international meetings to expand communication and use the scientific and economic capacities of international agencies and institutions.

## Introduction

In recent decades, the internationalization of higher education (IoHE) has seen an increasing trend, and the growth of international programs, expansion of branch campuses, joint partnerships, and increased global mobility of academics confirm this global approach to education (Hsieh, 2020; Galloway et al., 2020; Kudo et al., 2020). Higher education internationalization, as a strategic concept and agenda, is a relatively new, widespread, and diverse phenomenon in higher education (HE), evolving from fringe activities to an important aspect of the higher education reform agenda (de Wit & Altbach, 2021; Shahjahan & Edwards, 2022). IoHE has different meanings for experts and, as a result, there are several definitions of this concept. Altbach (2002) describes internationalization as the specific strategies and innovations of countries and educational institutions aimed at converging with global trends, including strategies for attracting international students, inter-university scientific partnerships, and the establishment of overseas academic branches. Knight (2003), in a comprehensive definition, states that “internationalization, at the national, sectoral, and institutional levels, is defined as the process of integrating an international, intercultural, or global dimension into the purpose, functions, or delivery of postsecondary education” (p. 2). Jones et al. (2021) emphasize the social responsibility of HE at the community and global levels to promote the common good. IoHE for society aims to benefit the wider community, at home or abroad, through education, research, service, and international or cross-cultural engagement (Brandenburg et al., 2019). In this approach, diversity in local and global communities is recognized and the dominance of specific priorities is not accepted; accordingly, internationalization activities are designed and implemented specifically to help solve local and global social issues.

IoHE is usually divided into two main categories: internationalization abroad and internationalization at home (Knight, 2004). *Internationalization abroad*, or cross-border education, relates to educational activities abroad and has a variety of types, including student or degree mobility, faculty/staff mobility programs, institutional mobility or branch campuses, and research programs abroad. Student mobility has always been one of the most popular forms of education in internationalization and is often regarded as its face (de Wit & Altbach, 2021; Stella, 2006). “*Internationalization at home* is more curriculum-orientated and focuses on activities that develop international or global understanding and intercultural skills” (de Wit et al., 2015, p. 45). This form of internationalization refers to “the purposeful integration of international and intercultural dimensions into the formal and informal curriculum for all students within domestic learning environments” (Beelen & Jones, 2015, p. 76). Other methods for internationalization at home include virtual and online learning and virtual mobility for joint courses and research projects, as well as hiring international scholars and academics (Knight, 2012). New information technologies, mass demand, pressure to commodify and commercialize knowledge, internationalization of professions, and migration of skilled labor all contribute to the unprecedented growth of borderless education. In contrast to single-mode programs, institutional dual-mode programs offer both in-person and online instruction. The dynamics of providing distance education have changed as a result of online education. Universities around the world have developed online programs since the 1990s to attract new domestic and international students. Universities, governments, donor organizations, publishers, and private businesses sponsor numerous networks and platforms with the intention of encouraging multi-level partnerships between research centers and universities. They have also created new opportunities for international scientific communication and networking, even in low-income nations that face significant challenges, such as inadequate infrastructure development, unfavorable policy and regulatory frameworks, and a lack of telecommunications professionals (Zeleza, 2005).

Internationalization is not a goal in itself, but a way to boost academic quality and development (de Wit, 2018). Economically, internationalization serves as a means of preparing individuals for work in a globalized economy, promoting national development and competition, and generating extra-institutional financial gain. Politically, internationalization promotes a much-needed understanding of peace and security in a controversial world and the development of worldwide citizenship. The internationalization of educational, research, and service activities of higher education institutions (HEIs) also improves its quality by requiring institutions to promote international academic standards (Knight, 1997; Kreber, 2009; Pherali & Lewis, 2019). International collaborations in higher education are incorporated into the academic goals and programs of many countries. Furthermore, governments and HEIs also try to develop their international programs in various ways. However, the level of IoHE varies across countries and regions. The level of income, the level of access to resources in HEIs, the quality of academic programs, institutional and national policies and programs, and various other factors affect IoHE in countries, each of which can be studied as a separate case (Ndaipa et al., 2022).

While admitting that research on IoHE has grown in recent decades throughout the African continent, particularly in South Africa, there is still a need for more studies on internationalization. To initiate more studies on this topic on low-income African countries, especially in East Africa, this study provides an overall overview of internationalization challenges that HEIs in these countries face in the process of internationalization. This is a study based on the critical analysis of existing documents and literature as well as semi-structured interviews. The findings contribute to the ongoing debate on IoHE in Africa in the post-pandemic era. Finally, based on the results of this study, strategies and measures are suggested to respond to the challenges of IoHE, which may be helpful for managers and academics at the institutional and national levels.

## East African higher education

The global movement of internationalization encompasses all regions, including Africa. In East Africa, after regional independence in the 1960s, HEIs sought to reinvent themselves and strive for global recognition. However, as a technologically backward low-income region, East Africa faced several problems in meeting both the national and global needs. Many universities in the region encountered obstacles in enhancing their teaching and research capabilities. Many institutions are currently significantly short of trained scientific staff, organizational infrastructure, research equipment, and sustainable power and internet availability. While the region suffers from brain drain to developed countries, institutions are expected to remedy problems related to development, destitution, and the provision of specialists such as medical doctors, professors, and engineers (Tan et al., 2021). Despite having access to most global structures and systems inherited from colonial powers and the more recent influence of donations, East Africa’s higher education system is probably the most marginalized within the world. Due to the influence of the colonial era, the university was built on the typical Western model of teaching first in English and then in Swahili, the official language of the region. However, challenges of English capability and the limited international presence of African scholars and students are well-known (Teferra, 2008).

In 1963, the Federal University of East Africa was established in Makerere, Uganda, as a result of the regional approach to HE in East Africa. The Federal University was formed by combining the three colleges of Nairobi, Makerere, and Dar es Salaam into university colleges. However, there was only brief cooperation in the provision of HE in the region. In 1970, political disputes in the region led to the collapse of the East African Community (EAC) and the federal university arrangement (Ogachi, 2009). Following regional policies, the Arusha Convention was formed under the leadership of UNESCO, committing member states to a long-term process of harmonization and recognition of HE qualifications and creating suitable conditions for professional and student mobility across African countries. In addition, the East African Higher Education Area was established to strengthen the international presence of East African HEIs and regional cooperation (Oanda & Matiangi, 2018). The Inter-University Council of East Africa (IUCEA) was then created to help maintain inter-university collaboration. The IUCEA acts as a regional coordinating and regulatory body to develop frameworks for regionalization of HE. For example, there is a transfer system to encourage increased student mobility and there are plans to increase staff exchange within the region and greater convergence in research and development initiatives in the region (Ogachi, 2009).

The process of IoHE in East Africa is developing. In less than 15 years, Kenya, Uganda, and Tanzania have seen an increase in the number of private HEIs, most of which are foreign-owned. Both private and public universities in the East African region have increased the number of joint agreements with international universities that facilitate student and staff exchange (Ogachi, 2009). Recently, the IUCEA and the World Bank signed a $10 million agreement to build greater capacity in the region to provide high-quality education and applied research in agriculture. The money will fund African agricultural HEIs in Malawi and Mozambique to strengthen agricultural HE and research in the region. In addition, as an example of partnership with international companies, in 2021, the Regional Center of Excellence in Information and Communication Technology in East Africa and IBM entered into cooperation to create digital skills for the EAC in several fields of education, promoting entrepreneurship, internships, and collaborative research and publications (IUCEA, 2022). During the Covid-19 pandemic, the University of Nairobi collaborated with the University of Cambridge on an oxygen concentrator and ventilator development project (UoN, 2020). Kenyatta University and the University of Girona have signed several agreements within a mobility scholarship in the framework of the European Union’s Erasmus + KA1 International Credit Mobility Project. The agreements have resulted in many approved grants allowing staff and students to travel between Africa and Europe (GTRCMC, 2021). In terms of educational assignments, the African staff exchange program is supported by the German Academic Exchange Program (DAAD). Such joint projects between East Asian HEIs and other international universities, carried out in the direction of scientific and economic development, have faced some challenges, however. The focus of programs offered by developed countries is sometimes not in line with the needs of Africa and is unable to provide a suitable solution to the problems of poverty and sustainable use of natural resources. There are also challenges in diversifying financing and quality assurance for establishing and continuing international partnerships (Ogachi, 2009).

Despite the fact that some international institutions have established campuses in the region and international programs are offered, students prefer to travel to the institutions’ home countries to study. This means that the flow of human and financial capital is always toward developed countries. Statistics show that Western countries are the main and first destination for students from the East African region (Ogachi, 2009). According to the data of the UNESCO Statistical Institute, of the five most populous countries in east Africa, Kenya had the highest number of outbound mobile students in 2020, at 14,060. Other countries in the region had fewer than 10,000 outbound mobile students. Kenya also hosted 6,828 international students, of which 6,186 came from other African countries and only 642 came from outside Africa; for other East African countries, the number is very small. The destination for most of the populous countries in East Africa is the USA, and of African countries, only South Africa is among the top five destinations for students from this region. The authorship trend shows that the first choice of East African academics in the field of research collaboration and publication were academics from North America and Western Europe (UIS, 2020).

Online and blended learning models have opened up new opportunities and issues for internationalization. Turning to online education and digitalization can provide several opportunities to academics, but doing so requires the necessary infrastructure and preparation. The International Association of Universities reports that only 24 percent of Africa’s population has access to the Internet, that there is poor or unstable connectivity in certain rural areas, and that many students cannot afford a device or data charges to connect to the Internet. During the pandemic period, additional costs had to be incurred by institutions, staff, and students for the transition to online education while research projects were halted, albeit for a short time, with students concerned about their academic future (Sonn et al., 2021). HEIs familiar with online education turned to it relatively quickly by using the requisite tools and teaching methods. By contrast, institutions that were much less prepared for online education had to work to improve the skills of their staff and students while requiring large investments in technology to fund the shift to online learning (du Plessis et al., 2022). Many lecturers, having little experience in providing online education, had to quickly become familiar with different systems to ensure the continuation of education online. Not all academics had personal computers or laptops and proper Internet access at home. In some cases, laptops had to be shared with spouses or children, who also needed them at home (Hedding et al., 2020). According to the International Association of Universities, Africa has the largest number of closed campus universities due to COVID-19 compared to the USA, Europe, Asia, and Oceania. Africa is also the only region where education has been suspended or discontinued for a period of time before switching to online education and learning. Only 29 percent of HEIs in Africa adapt quickly to online education, compared to 85 percent of HEIs in Europe, 72 percent in the USA, and 60 percent in the Asia Pacific region (Sonn, et al., 2021). The COVID-19 pandemic further exposed educational inequalities, while some institutions prepared to move toward online education and resume classes while others confronted intense restrictions related to lack of access to technology and poor socioeconomic conditions among students (du Plessis, et al., 2022).

The internationalization opportunities for less developed countries include targeted and sustainable interactions at the global level and partnerships with associations and international agencies, as well as using the capacities of the Sustainable Development Goals (SDGs). The SDGs place a special emphasis on education, with its fourth goal stipulating that all learners should have the same access to quality education and the right facilities for the same throughout their lifetime. In the absence of cooperation with other countries, especially those with successful experience in this field, these goals will be difficult to achieve (Heleta & Bagus, 2021). Hence, Target 4.c and 7.a states:

By 2030, substantially increase the supply of qualified teachers, including through international cooperation for teacher training in developing countries, especially least developed countries and small island developing States” and “By 2030, enhance international cooperation to facilitate access to clean energy research and technology. (United Nations, 2015, pp. 17,19)

Also, Target 17.6 under global partnerships stipulates:

Enhance North-South, South-South and triangular regional and international cooperation on and access to science, technology and innovation and enhance knowledge sharing on mutually agreed terms, including through improved coordination among existing mechanisms, in particular at the United Nations level, and through a global technology facilitation mechanism. (United Nations, 2015, p. 26)

In consistence with the SDGs, multiple collaborations have been established between higher education institutions (HEIs) in the high-income Global North and its low-income southern partners to create or enhance the research capacity in the latter (Horwood, et al., 2021). Therefore, African countries, especially the least developed ones in East Africa, can benefit from international endeavors to develop higher education and increase academic partisanships internationally, in accordance with the SDGs.

## Research methodology

The aim of this study is to discover the challenges faced by low-income African countries, especially focusing on East Africa, in the process of IoHE and to further provide strategies to respond to them. For this purpose, due to the exploratory nature of this research, qualitative research methods which rely on in-depth data analysis were used (Corbin & Strauss, 2013). The case study was conducted using data sources obtained from semi-structured interviews and documentary analysis aimed at answering the research questions (Creswell & Clark, 2011). In order to collect data, 10 interviews were conducted online. The purpose of the research was discussed with the interviewees, who ensured that their participation was voluntary. With their permission, the conversations were recorded so that the information could be transcribed with high accuracy and also reviewed, if necessary. For anonymity and confidentiality, the identities of the participants were protected in this research using pseudonyms.

### Sampling and participants’ profile

In this study, a purposive sampling technique was applied. Purposive sampling is used to reach people who have gained in-depth knowledge of a subject as a result of their professional role, experience, or expertise (Cohen et al., 2011). Therefore, to meet the purpose of this study, we targeted academics in low-income African countries, especially in East Africa. We purposefully selected academics who have published papers on “Higher Education” and “Internationalization” in collaboration with international partners, which are indexed in science databases such as Web of Science, Scopus, and JSTOR. With this criterion, we made sure that the interviewees firstly had the experience of international participation and, secondly, were familiar with the literature on the subject. This search resulted in a list of 30 academics in universities in East Africa. We then invited them for interview; 10 accepted the invitation. The profiles of the participants, in terms of their gender, education, and location, are presented in Table 1.

**Table 1:** Profile of the participants

Title of the participant	Sex	Education	University	Country
P1	Male	PhD	Moi University	Kenya
P2	Male	PhD—Assistant Professor	University in Kakamega	Kenya
P3	Male	PhD—Assistant Professor	University in Addis Ababa	Ethiopia
P4	Male	PhD—Assistant Professor	Addis Ababa University	Ethiopia
P5	Male	PhD-Senior Lecturer & Chair	Moi University	Kenya
P6	Male	PhD—Associate Professor	Kenyatta University	Kenya
P7	Male	PhD—Associate Professor	Addis Ababa University	Ethiopia
P8	Female	PhD—Associate Professor	Busitema University	Uganda
P9	Male	PhD—Senior Lecturer	Uganda Management Institute	Uganda
P10	Female	PhD—Senior Lecturer	University of Nairobi	Kenya

### Data collection, analysis procedure, and ethical considerations

A flexible interview guide was used for data collection which enabled the researchers to customize questions depending on the interviewee’s role and experience, as is recommended for case studies (Ozcan et al., 2017); in addition, some were followed by probing questions to clarify meaning and capture responses greater detail. The key semi-structured questions in the interviews were about (i) the challenges and obstacles to the internationalization of African universities (low-income countries) at three levels of international, national, and institutional, (ii) the strength of universities in the country for participating in international academic activities, (iii) opportunities for internationalization, and (iv) impact of COVID-19 and pandemic and SDGs on internationalization activities of universities. In addition, documentary analysis included the study of research, articles, and reports on IoHE in Africa, especially in low-income East African countries. The recorded interviews were transcribed and checked for accuracy. Thematic analysis was then deployed to analyze the data. Following Braun and Clarke (2006), the initial codes were first extracted. These related to the informants’ experiences of issues and challenges they had observed or experienced in international activities or collaborations. Similar codes referring to a common concept were then placed next to each other, and unrelated codes were removed. For each of the challenge categories, a suitable label was chosen to represent their concept. These challenges included a wide range of issues, ranging from the cultural and social to the financial and infrastructural. For this reason, in the last stage of the analysis, taking into account the purpose of the study and the nature of the challenges, more comprehensive categories were formed and the final framework was produced. The documentary analysis data were also compared with the interview results and amended or supplemented, as needed. Finally, the output was displayed in the form of specific categories while citations and evidence were examined to confirm the insights gained. To ensure the validity and reliability of the results, the following measures were taken (Riege, 2003; Brink, 1993): All interviews and data were carefully coded using MAXQDA 2020 software. To resolve any ambiguity, two new questions were sent to the interviewees and their comments were taken into account. The output of the analyses was presented in five sessions to three external researchers and their opinions were documented. All the stages of this study, from literature review to data analysis, were reviewed by the second and third researchers and possible corrections were implemented. Finally, the output was sent to the interviewees and their criticism and suggestions were examined. It should also be noted that to find out the latest opinions and studies on the internationalization of higher education, researchers participated in the following three events and conferences and used the views and information expressed to enrich the study: UniPID Webinar 2021 (How to foster responsible and fair global academic partnerships), SDG Conference Bergen 2022 (Higher education and sustainability challenges in the 2030 agenda), GINTL Launch Event 2022 (Sparking Higher Education Partnerships for Education Development), and the 20th Triple Helix Conference 2022 (Universities connecting local communities and global knowledge networks).

## Findings and discussions

**Fig. 1:**
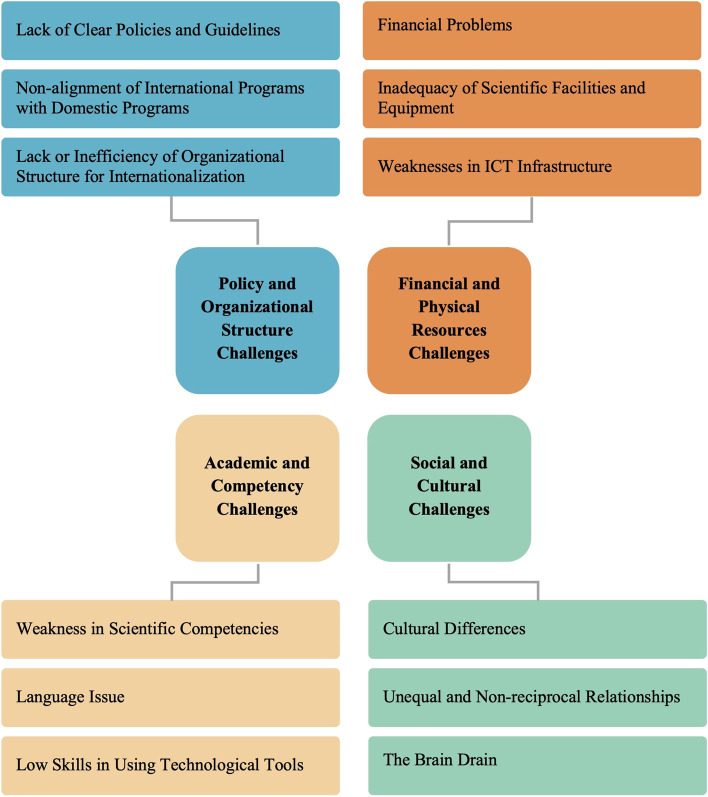
Challenges of internationalization of higher education

After analyzing 10 interviews, 154 initial codes were obtained and the proposed model for the challenges of IoHE was formed after thematic analysis, according to the semantic commonalities of the codes. These challenges were divided into 4 main categories and 12 factors, as depicted in Fig. 1.

### Policy and organizational structure challenges

#### Lack of clear policies and guidelines

The opinions of many interviewees showed that one of the challenges for many African universities is the lack of a specific, precise, and clear plan for internationalization. A Nigerian university professor stated:

I think, from where I sit, policy guidance is what could be a challenge because in our situation, the policy framework that would guide activities of internationalization, it is not very specific. (P5)^1^

The interviewees reported that in some universities, internationalization has not received much attention in general, and there is not even a plan and vision for the same. In other institutions of higher education, many internationalization programs are incomprehensible, too generalized, and fail to determine the role of each component of the institution for this purpose. This issue is also observed at the national level. An East African university professor pointed out:

You cannot imagine where it will take you when you don’t have strategies, when you don’t have policies, when you don't have directives. So, at the national level, it’s a very, very delicate one. (P7)

For this reason, ambiguity and weakness in policies and regulations, both at the national and university levels, have become obstacles to IoHE in Africa. The policies are neither clearly defined nor does its academic components work together effectively. Studies by Tamrat and Teferra (2018) on Ethiopian HEIs have also addressed this issue. They state that data obtained from literature sources and group discussions display a clear lack of internationalization strategies and policies at the institutional and national levels, which is why the institutions’ efforts for the same do not yield the expected results.

#### Non-alignment of international programs with domestic programs

Another challenge faced by this sector is the lack of alignment of the domestic programs of universities with international ones. In some universities that have had international partisanships, the areas of participation and activities are not compatible with the domestic programs and the needs of African universities and society. Thus, the outcomes of such partnerships do not substantially benefit the African partner. This can have a variety of causes, of which the most important one is the lack of strategic plans and policies that cause universities to engage in international collaborations only to attract resources or other internationalization privileges. A university professor from East Africa said:

Sometimes, you see them engaging themselves in activities or implementing something which was not originally planned. It’s only because there was a partner which is interested to do something which was not originally the interest of an African university. (P7)

One of the most important functions of universities is to meet the needs of the country and its local environment, which will subsequently contribute to local development. Therefore, research topics and international activities will be more effective if they are in accordance with the above needs. One of the professors insisted:

We must work with a global perspective, taking into account local challenges, local contacts, and try to see how we can provide local solutions to those challenges. (P8)

African universities continue to implement largely alien academic programs and roles, even after the Africanization of some curricula. However, the output of African higher education is not yet consistent with the development of African communities and does not address their basic needs (Phuthi, 2022). The participants emphasized that research topics and international activities should be defined in a way that can give hope to Africa and match its needs and problems.

#### Lack or inefficiency of organizational structure for internationalization

The organizational structure of universities should be formed in accordance with internationalization strategies. The interviewees emphasized that there is no organizational structure for international communication in some universities while many existing structures are inefficient and have been unable to play a major role in the internationalization of universities.

Most of these institutions don't have an office of international relations where they can connect with other institutions outside. So in the process they cannot keep any proper data of visitors who come, who wants to cooperate with them, who wants to work together with them. (P10)

Another interviewee indicated:

we need to establish international offices with people or staff who are well trained to engage with different activities, including recruitment of international students, managing international projects, different welfare issues for international students, which is actually not the case in most of the African universities. (P1)

The existence of an international office or department in universities and the coordination of decisions and actions among all its subordinates are essential. International activities and partnerships have different dimensions that require handling by specialized professionals. The limited capacity and effectiveness of offices established to manage and promote internationalization are common in Ethiopian HEIs. These flaws are mainly reflected in the way internationalization efforts are initiated, directed, and controlled. Most institutions are far from implementing systematic internationalization processes (Tamrat & Teferra, 2018).

### Financial and physical resources challenges

#### Financial problems

The analysis of internal and external obstacles to internationalization clearly highlights the importance of financial resources. “Inadequate funding” is one of the main obstacles in Africa and elsewhere. Most African universities are funded by the government and economic problems pose a serious threat to the development of educational activities (James & Xiong, 2020). Many African HEIs face the challenge of inadequate national funding, and the resources and budget allocated to promote international activities are meager (Heshmati & Hartvigson, 2018; Owusu-Agyeman, 2021; Tamrat & Teferra, 2018). This is one of the key factors emphasized by all participants,

Funding is a challenge not only to internationalization alone, but to the entire higher education sector itself. (P1)

Many African universities are dependent on government funding; therefore, the government’s economic problems have a direct impact on academic performance, as pointed out by an African professor,

Most of the universities that are on the continent are public universities and they depend on the government for their funding. (P2)

The lack of financial resources has changed the priorities of many universities and led them to focus more on domestic activities. As stated by one of the interviewees,

In many countries, their focus is on providing higher education to their own citizens. Internationalization is a luxury for many governments so they don’t really prioritize it. They try to provide equitable higher education to their citizens, for example, in Ethiopia, building institutions, infrastructure, regional regions, and etc. (P3)

#### Inadequacy of scientific facilities and equipment

Students and professors need access to new scientific facilities and equipment to develop their knowledge and experience. This is crucial for the development of their capability to cooperate and compete internationally with universities that benefit from these facilities. This is one of the reasons for the academic weakness of African universities, preventing them from collaborating on advanced research topics across the world. A university professor from Kenya said:

when we talk of education internalization, we are talking of infrastructure. What infrastructure do we have so that these students can be compared within the global context? So, there is lack of adequate structure. (P10)

In many HEIs, research equipment and institutional infrastructure are significantly lacking, and electricity and internet connectivity are not sustainable (Tan et al., 2021), as proclaimed by a respondent of this study:

you do not have adequate laboratory facilities or adequate equipment to exploit the potentials that you have. So the institutional infrastructure is another challenge for the academic staff. (P1)

Having up-to-date equipment in laboratories is an important factor for the scientific growth of universities and can be an advantage for the expansion of their international partnerships. Limitations in laboratory equipment hinder advanced research, which also leads to frustration (Jamison & Madden, 2021).

#### Weaknesses in ICT infrastructure

In today’s world, many universities offer services using new technologies, especially through virtual platforms. This became even more apparent during the COVID-19 pandemic. Many educational institutions were forced to resort to e-learning while several international collaborations, research, conferences, and seminars were virtualized. Online access opportunities in the information age is the first step towards IoHE (Yıldırım et al., 2021). Given the concept of internationalization at home, many international activities can take place virtually. Using the educational content of the world’s best universities through virtual platforms and virtually participating in international conferences are examples of the application of the Internet and information technology in internationalization. In Africa, one of the most critical issues is the weakness of ICT infrastructure. A university professor from Kenya remarked:

Getting infrastructure for digitalization is still an issue. I want to say you’ll be surprised that the network that I could have in my house now is likely to be a much better network than what I would get at my university ... at university, we share with so many people, so you need broad bandwidth, which is not easy to get, because of the cost. (P5)

Another university professor commenting on online education in African universities said:

Many African universities were not able to continue their undergraduate and graduate programs online because of the poor infrastructure that they have. (P7)

According to the opinions of the interviewees, this issue received more attention during the COVID-19 pandemic and has led to investment in infrastructure development. However, it still remains a serious challenge, especially for areas far from urban centers, as one respondent remarked:

We still have dark spots in terms of access to the internet and the divide between rural and urban, rich and poor is still there when it comes to access to technologies. So yes, the terrain has improved, but there is a lot more that would need to be done. (P8)

### Social and cultural challenges

#### Cultural differences

Research findings show that although cultural differences can be an opportunity for innovation, they can sometimes also be challenging to communication. The impact of internationalization on regions, countries, and institutions varies according to specific contexts (de Wit & Altbach, 2021). Successful international collaboration requires both partners to recognize each other’s expectations and values and to have a deep understanding of how local laws and regulations condition the opportunity to expand collaboration. These dynamics are essential for long-term partnerships (Balbachevsky et al., 2020). A university professor from Kenya stated:

Cultural differences can actually be a major impediment, and for projects of collaboration to work very well, there will certainly be a need for some cultural orientation before one gets very comfortable. (P5)

Throughout the process of internationalization, they must analyze the local realities in which they operate, that is, their cultural origins and traditions, which can vary greatly across countries and have an undeniable impact on IoHE. Thus, it is also important to pay attention to the perception of institutional actors about internationalization (Sá & Serpa, 2020). It was also emphasized in the interviews that intracultural integrations should not overwhelm a particular culture, stressing the need to understand the context and conditions of Africa in the sense of internationalization.

In most cases, scholars or the management body in African universities are trying to understand internationalization in terms of the Western scenario. We are not well aware of the benefits and dangers of internationalization in terms of the circumstances and contexts of the universities in Africa. (P4)

Pherali and Lewis (2019) emphasized that there are pertinent concerns about North–South participation, given the potential dominance of Western educational values in the capacity building process. Also, a number of cultural divisions and differential understanding of research affect the perception of power and equality in all aspects of such relationships. Cultural differences affect international interactions among faculty and students, both in teaching and research. According to the interviewees, people sometimes have expectations that have a different meaning in a different culture, which may lead to misunderstandings. A university professor from Uganda stressed,

There is little understanding about the challenges that students were having by integrating themselves into the university setting out there. So, I think it requires a little more orientation, a little more preparation of both the academic staff and the students for that mobility to work out well. (P8)

#### Unequal and non-reciprocal relationships

Data analysis shows that unequal relations are another major challenge in the internationalization of African higher education. Asare et al. (2022) stated that in international partnerships, with a few exceptions, countries with less developed research capacity benefit the least, emphasizing that the source of control at the start of a project has a key impact on equality in relations. Budget conditions of the Global North tend to strengthen the “donor-recipient” relationship, which puts southern researchers under the leadership of the North. This inequality is more evident in the benefit of the results of partnerships. One of the participants in this study emphasized:

Whenever African faculty institutions collaborate with European and American institutions, the resources mostly are from them, so we rely on resources available in other continents, which also limits African researchers and faculty’s ability to influence the nature of the projects and research. So, it’s always the Americans and the European’s research objectives that are really implemented. (P3)

Another East African professor affirmed:

They say the partnership is on equal foot, but that’s not the reality on the ground. Those who finance that kind of, let’s say, if it’s a mobility, a research partnership, or a joint master’s program, those who finance would have much more say than those who seek funding for that partnership and your power to set agendas would be also limited at the institutional level. (P7)

Students’ motivations also vary, as a professor at an African university noted:

They (non-Africans) want to do things in the communities to researchers, but very few would attend courses, get a certificate from the African universities that they collaborate with. They mainly would like to work with communities on health, on water. But African students going to Europe or America will attend a class, will get a certificate and these are attitudes, perceptions about the quality of programs, learning environments and etcetera. (P1)

It is important to note that the financier of projects and programs usually determines the issue, which creates inequality since the other partner is unable to play a decisive role. As one participant indicated,

Building that capacity becomes problematic when leadership for most of the schemes is coming from the North and the researchers in the African institutions do take on roles, but not roles that are to do with managing the research. (P8)

The dominant system of internationalization treats knowledge as a commodity for sale and consumption, prioritizes economic growth, globalizes Western knowledge, and makes Western-led human progress and development the ultimate goal (Stein & Silva, 2020). It is necessary to prevent excessive strengthening of this imbalance and unilateral leadership by providing new frameworks and strengthening South–South relations (Pherali & Lewis, 2019).

#### The brain drain

Human capital is an effective factor in the economic growth and development of countries. The challenge of “brain drain,” or the departure of talented professionals and individuals from the country and their non-return, is an important issue in Africa. Many African countries suffer from the loss of their skilled human resources in various fields of science. This has had a terrible impact on the sustainable development of these countries (Mlambo & Adetiba, 2019). According to participants, brain drain could be one of the negative aspects of internationalization, especially for low- and middle-income countries. A university professor from Ethiopia noted:

Internationally, the engagement is not on equal basis and balanced. It has become a means of brain drain for Africa and brain gain for the West. (P4)

A participant from Dakar further added:

People are leaving, going outside and not coming back, or external funding agencies are locating research outside the universities and, therefore, borrowing out capacity from within the universities. They’re targeting individuals, pretty well-trained individuals. (P6)

### Academic and competency challenges

#### Weakness in scientific competencies

The quality of education and academic programs as well as the academic abilities of professors and students is important for both teaching and joint international research. Despite efforts to develop higher education in Africa, its efficiency and adequacy are comparatively lesser than the demand and lacking in effectiveness and alignment with the average international standards (UNICEF, 2021). International universities are reluctant to connect with institutions having poor research capabilities. Similarly, international students prefer studying at a university that is recognized for the quality of its teaching and research and can compete internationally. According to the participants, African higher education does not have a high capacity to compete internationally. There are several reasons for this, as one respondent mentioned:

For a number of colonial and national reasons, most African academics are either not well qualified or interested in their professional and academic performances. This problem is further complicated by brain drain. Also, the instructional media or the languages used to teach and learn the programs, in most cases, are alien to the African community or they are colonial. (P4)

Commenting on the individual and academic capabilities of African universities, a university professor from Uganda stated:

Africa has some seasoned researchers, some of whom were trained in the Global North and whose research has made an impact globally. But the capacity to participate in research is low since most HEIs in Africa are teaching-intensive. Within African higher education, joint international research is viewed as a strategy for building the capacity of researchers and this attests to weak capacity. (P9)

Many African universities are struggling to improve their teaching and research capabilities. In many institutions, there is a critical shortage of qualified scientific staff (Tan et al., 2021). Therefore, despite the high potential of African students and professors to participate in international activities, low research and education capacity in universities coupled with the lack of opportunities have hindered their prosperity and the development of African higher education internationally.

#### Language issue

Language is one of the primary requirements for international interaction. The lack of proficiency in international languages among students and professors can be an important challenge for African countries. Studies by Pherali and Lewis (2019) in Somaliland emphasize that in an international project, their limited English capability considerably prevented them from expressing their thoughts in writing and also limited them academically. A university professor from Kenya stated:

The other issue that you cannot again ignore on the African continent is the language factor. It may look marginal but that is crucial. (P2)

A university professor from Uganda, referring to the numerous higher education systems in Africa and educational languages, said:

Language can also be a barrier to internationalization. Somebody from Anglophone Africa, it is hard to get a placement in a Francophone country. So, the multiple higher education systems in African countries are a barrier to or a challenge to internationalization. (P9)

Despite offering English programs, there are challenges with regard to English proficiency among academics and students which prevents them from getting involved in the international arena (Tan et al., 2021).

#### Low skills in using technological tools

Many African universities have not been able to equip themselves with new technological tools and facilities due to their lack of resources and economic problems. As a result, many of their students and teachers do not have the necessary skills ratio in using some of these tools and equipment. During the COVID-19 era, when many universities were forced to resort to e-learning, it was also observed that some students had difficulty using these platforms and their related tools. One professor pointed out the challenge faced by students while using these platforms:

It’s not that every student understands how to use technology. But we are supposed to use technology, and so technology is a huge challenge but is the way to go. (P2)

Some have considered the use of new technologies for learning and research as an important change and, as often stated, there is always resistance to change. However, over time, people have been acquainting themselves with these new methods and tools. The COVID-19 pandemic has been a turning point in the digitalization of higher education. A university professor from Ethiopia stated:

Now gradually, they (institutions) are learning how to provide online education. Students also have limited capability, as you know, they have a number of challenges—social, economic, political challenges. So, when you add these challenges to their daily lives, it’s frustrating. But gradually and slowly, they are learning how to use online teaching platforms. (P3)

In general, the process of digitalization of higher education requires a change in the overall culture of the educational environment and the growth of stakeholders’ digital literacy (Laufer, et al., 2021).

## Conclusions and proposed strategies

The present study sought to identify the challenges of IoHE in low-income African countries, mainly in East Africa. Challenges were identified in four major categories: policy and organizational structure, financial and physical resources, social and cultural, and academic and competency challenges. The results confirm many of the challenges noted in the literature and place further emphasis on financial issues, the importance of sound information technology infrastructure, and the dominance of Western hegemony, which is largely the legacy of colonialism. Now that the COVID-19 pandemic era is coming to an end and there is little time remaining until the deadline for the SDGs, it is crucial to take effective measures to accelerate IoHE. To this end, measures and strategies that can respond to these challenges are suggested. The strategies were categorized based on the internal and external stakeholders of the institutions to determine their roles in promoting IoHE in Africa.

### Internal strategies, policies, plans, and organizational structures

HEIs should develop transparent policies and visions, along with both short-term and long-term plans, and inform all academics and employees about the same. Being transparent and having detailed programs prevents confusion and helps determine the role of different stakeholders. It is necessary to note that professors and students need incentives to participate in international activities, which is why regulations need to be developed according to existing capacities. Conditions should be facilitated for academics to easily attend meetings and conferences as well as join associations and consortiums to form a global sustainable communication network. It is very important that the responsible unit for international interactions and activities, in the organizational structure of institutions, is clearly specified and its duties and powers are precisely defined. This unit can also play an influential role in information and knowledge management by navigating and monitoring the supply and demand for international interactions.

### Internal strategies, human resources

Human resources are the most important pillars of any organization, more so in universities for academics to keep their scientific information and skills updated to be internationally effective. Various measures can be helpful for this purpose, such as attending international conferences and seminars in person or online, membership in international forums and associations, and continuously studying the latest articles related to their fields. Another requirement of effective interaction at the international level is for the development of intercultural communication competencies. Academics need to develop their skills to properly interact with people from different cultures and also use new technologies in education, research, and teaching.

### Internal strategies, finances, physical resources, and technological capabilities

The importance of financial resources for international activities cannot be overstated. HEIs should allocate specific funding for the development of international communications, publications, recruitment of foreign students and professors, support for students and professors to participate in courses, international conferences and events, and other activities that could contribute to greater engagement in the international academic atmosphere. Also, institutions and universities should be able to equip themselves with updated facilities and new technologies. This would encourage and enable international competition and cooperation and can also improve the level of collaboration within the global academia.

### External strategies, political-legal

Beyond HEIs, while the government is the most important factor, other organizations and foundations also play important roles in the development of international scientific activities. At the macro level, governments should develop practical policies in dealing with institutions and universities, while considering both contextual and environmental conditions in their planning. Avoiding centralism and granting autonomy to universities can greatly facilitate the establishment of international connections. Concluding international agreements with different countries, ensuring the quality of degrees for admission by other countries, introducing Africa’s capacities at international meetings, and actively pursuing the UN programs (such as SDGs) are some of the opportunities for IoHE. Governments should also facilitate the visa application process for international academics in Africa and improve other facilities needed for their comfortable attendance, such as finances, language, and accommodation. The African continent itself is highly capable and different regions can meet each other’s needs in terms of resources and experts. For this reason, establishing regional centers to synergize capacities to establish a stronger presence in the field of internationalization can be effective.

### External strategies, economic

Improving economic conditions has a direct relationship to the development of international higher education activities. Governments should commit themselves to allocating sufficient funds for internationalization and put their efforts into attracting investment for higher education in Africa from developed countries, in cooperation with other international funding agencies. Paying attention to the improvement of social services and welfare of international academics is another issue related to this field. Due to the increasing growth of virtual interactions, the development of ICT infrastructure has become more crucial than ever.

## Limitations and future research

Like other studies, this one is not without its limitations. The findings are built on the views of academics from HEIs in East Africa with research experience in HE internationalization. Future studies can advance the proposed model by testing it with other types of informants such as deans of HEIs, senior and junior faculty members in different fields of studies and research, and even administrators, students, and researchers engaged in internationalization initiatives. In addition, the model could be tested by qualitative methods or survey studies in countries in Africa or other developing countries. Empirically understanding the association or causality among challenges could present another opportunity for future research, which might provide insights for managers into priorities and locations for the investment of their limited internalization resources. It is also suggested that future studies explore the role of government and national policy-making institutions in IoHE in increasing or mitigating the challenges of IoHE.

In this study, the scope of IoHE is broad, and we did not explore specific internationalization programs. Our main goal was to collect the opinions and experiences of a group of informants and combine them with collected secondary data to offer an overview of internationalization challenges that HEIs in East Africa face in the process of internationalization and, ultimately, to spur more studies on HE internationalization at HEIs in East Africa. Focusing on understanding the outcome or implementation challenges of each of the different forms of internationalization abroad, such as franchise operations, branch campuses, articulation and twinning programs, and joint and dual degree programs, which are gradually being implemented in some African universities, will be valuable for policy makers and HEI managers. Using methods such as action research would be useful for understanding the extent to which proposed strategies to mitigate the challenges of IoHE are feasible and useful for HEIs in East Africa.

Moreover, this study used a grounded theory to collect and analyze data. Future studies could build on the proposed model and consider theories to explain why some HEIs succeeded in internationalization programs, or they could empirically investigate cost–benefit analyses or the outcomes of internationalization adoption at HEIs in East Africa in terms of quality, access, equity, or cost of HE services.

## Data Availability

The transcripts of the interviews are reserved by the authors and have been archived by the Tampere University. Due to the confidentiality of the interviewees’ information, the names of the individuals have not been released.

## Footnotes

1 Pn refers to quotes extracted from interviews and “n” is the participant number.
